# Tolerance Associated Gene Expression following Allogeneic Hematopoietic Cell Transplantation

**DOI:** 10.1371/journal.pone.0117001

**Published:** 2015-03-16

**Authors:** Joseph Pidala, Gregory C. Bloom, Steven Eschrich, Minnie Sarwal, Steve Enkemann, Brian C. Betts, Francisca Beato, Sean Yoder, Claudio Anasetti

**Affiliations:** 1 Blood and Marrow Transplantation, Moffitt Cancer Center, Tampa, FL, United States of America; 2 Bioinformatics, Moffitt Cancer Center, Tampa, FL, United States of America; 3 Department of Surgery, UCSF School of Medicine, San Francisco, CA, United States of America; 4 Molecular Genomics, Moffitt Cancer Center, Tampa, FL, United States of America; Beth Israel Deaconess Medical Center, Harvard Medical School, UNITED STATES

## Abstract

Biologic markers of immune tolerance may facilitate tailoring of immune suppression duration after allogeneic hematopoietic cell transplantation (HCT). In a cross-sectional study, peripheral blood samples were obtained from tolerant (n = 15, median 38.5 months post-HCT) and non-tolerant (n = 17, median 39.5 post-HCT) HCT recipients and healthy control subjects (n = 10) for analysis of immune cell subsets and differential gene expression. There were no significant differences in immune subsets across groups. We identified 281 probe sets unique to the tolerant (TOL) group and 122 for non-tolerant (non-TOL). These were enriched for process networks including NK cell cytotoxicity, antigen presentation, lymphocyte proliferation, and cell cycle and apoptosis. Differential gene expression was enriched for CD56, CD66, and CD14 human lineage-specific gene expression. Differential expression of 20 probe sets between groups was sufficient to develop a classifier with > 90% accuracy, correctly classifying 14/15 TOL cases and 15/17 non-TOL cases. These data suggest that differential gene expression can be utilized to accurately classify tolerant patients following HCT. Prospective investigation of immune tolerance biologic markers is warranted.

## Introduction

Biologic markers of immune tolerance may facilitate individualized management of immune suppression following transplantation. While experimental evidence supports multiple active cellular and molecular mediators of immune tolerance,[1] less data exists in the human clinical setting following solid organ or allogeneic hematopoietic cell transplantation (HCT). Clinical transplantation tolerance has been characterized by absence of ongoing immunologic injury due to incompatibility between donor and recipient without ongoing immunosuppressive (IS) therapy. Acute and chronic graft vs. host disease (GVHD), the major clinical manifestations of immunologic injury after HCT, commonly develop or reoccur after attempted IS discontinuation and result in morbidity and mortality.

Investigators have reported differential gene expression associated with the tolerant clinical phenotype in solid organ transplantation.[2–5] At the present time, clinical application is limited, as the majority of solid organ transplant recipients require life-long immune suppression. Conversely, while most patients eventually discontinue IS after HCT, current scientific understanding is limited: Data on the required duration of IS after HCT is largely lacking.[6] Clinical judgment does not discern drug-suppressed immune response from development of immune tolerance, and there are no validated clinical or biologic determinants of immune tolerance after HCT. Thus, current practice of IS discontinuation after HCT is empiric, markedly heterogeneous and complicated by GVHD following IS discontinuation.[7]

Insight into mechanisms of clinical transplantation tolerance and translation of this knowledge to strategies for individualized management of IS would represent major advances. We examined peripheral blood immune cell subsets and differential gene expression between tolerant patients after HCT, non-tolerant HCT patients, and healthy control subjects to discover biologic markers of immune tolerance.

## Patients and Methods

### Identification of study patients

From long-term survivors of allogeneic hematopoietic cell transplantation (HCT) in the Moffitt Cancer Center Blood and Marrow Transplantation Program, tolerant patients (TOL) were identified. Healthy volunteers were recruited to serve as control subjects. Demographic information (age, gender) was collected, and volunteers completed a brief medical questionnaire to confirm they were not acutely ill, had no chronic medical conditions and were not taking medications. These healthy control subjects were of interest, as they had not received HCT and were not treated with IS.

### Tolerant and non-Tolerant clinical phenotype

The tolerant phenotype was defined by successful discontinuation of all IS agents (minimum time from complete discontinuation of IS to time of sample acquisition of 6 months), and sustained absence of any detectable clinical, radiographic, or laboratory manifestations of acute or chronic graft vs. host disease. The absence of manifestations of graft vs. host disease was confirmed by at minimum two transplant physicians in each case. We acknowledge the lack of a robust standard clinical definition for tolerance post-HCT, however report here the sustained absence of GVHD among TOL cases in this series on long-term follow up. Through systematic search of the program database including all allogeneic transplant recipients, matched non-tolerant comparators (non-TOL) were identified who were not able to discontinue immune suppression due to GVHD. Non-tolerant comparators were matched to the individual tolerant cases by date of HCT (+/- 6 months) and age at time of HCT (+/- 5 years). From all possible non-tolerant comparators for each case, the best matched non-tolerant comparator was selected according to identity on the following factors in descending rank order: HLA matching between HCT donor and recipient (identical at HLA-A,-B,-C, and—DRB1 vs. mismatch), donor relation (sibling vs. unrelated donor), stem cell source (peripheral blood vs. bone marrow), GVHD prophylaxis agents, disease requiring transplantation, and conditioning regimen. This study was approved by the University of South Florida Institutional Review Board. All subjects provided written and verbal informed consent to participate in the study.

### Assessment of clinical data

For all tolerant and non-tolerant HCT recipients, standardized medical record abstraction was performed. Baseline demographic and transplantation variables included the following: age at time of HCT, condition requiring HCT, remission status at time of HCT, stem cell source, CD34+ cell dose/kg body weight, donor relation, donor age, gender matching of donor and recipient, HLA matching at HLA-A,-B,-C, and—DRB1 loci, cytomegalovirus serologic matching between donor and recipient, conditioning regimen, and GVHD prophylaxis agents utilized. Comprehensive information was gathered on prior manifestations of acute and chronic GVHD including the following: Initiation and discontinuation dates of all immune suppressive agents; onset, peak grade, biopsy confirmation, therapy delivered, resolution date, and recurrent manifestations for both acute and chronic GVHD according to consensus criteria.[8,9] Additional outcome data included relapse, death, last clinical follow up, and discontinuation of all IS.

### Sample processing and cell subsets

Each subject consented to peripheral blood collection, which included two 10cc EDTA tubes. Freshly acquired samples were immediately processed uniformly in a single center using standard operating procedure, and analyzed in one batch. From one sample, peripheral blood mononuclear cells (PBMC) were isolated using the Ficoll-Hypaque method, and were immediately processed for characterization of cell phenotype by flow cytometry. PBMC were stained with labeled antibodies: T cells (CD3-Percp5.5, CD8αβ-FITC, CD8αα-PE, CD4-Alexa700, CD25-PE-Cy7, CD127-Alexa647); NK, B cells, and monocytes (CD3-Percp5.5, CD16-Alexa700, CD56-PE, CD19-PE-Cy7, CD14-FITC); Dendritic cells (HLA-DR-Percp-Cy5.5, Lin1-FITC, IL-3Ra (CD123)-PE, CD11c-APC). All antibodies were from BD Biosciences, except live/dead-yellow (Invitrogen). Red blood cells were lysed, samples washed, and samples were analyzed using the LSR II flow cytometer (BD Biosciences). We quantified immune cell subsets according to the following phenotypic markers: total CD4 T cells (CD4+); total CD8 T cells (CD8+); αβ CD8 T cells (CD8+, αβ TCR+); αα CD8+ cells (CD8+, αα TCR+); regulatory T cells (CD4+,CD25+,CD127(low)); NK cells (CD16+, CD56+); B cells (CD19+); Monocytes (CD14+); type 1 Dendritic cell (HLA-DR+, CD11c+, Lin-); type 2 Dendritic cell (HLA-DR+, IL-3Rα+, CD4(low), CD11c-, Lin-). Due to multiple comparisons, we utilized a pre-defined level of significance (p < 0.01) for comparisons between groups.

### RNA extraction and microarray analysis

PBMC were similarly isolated from the second sample, and total RNA was extracted to serve as the mRNA source for microarray analysis. RNA extraction was performed using the RNAeasy Mini Kit (Qiagen), and RNA was quantified using a NanoDrop 1000 spectrophotometer. The RNA quality was assessed using an Agilent 2100 Bioanalyzer. The poly(A) RNA was converted to cDNA, then amplified and labeled with biotin following the procedure initially described by Van Gelder et al.[10] Hybridization with the biotin labeled RNA, staining, and scanning of the chips followed the procedure outlined in the Affymetrix technical manual.[11] All analyses used the Affymetrix Human U133 plus 2.0 array, which contains approximately 48,000 probe sets designed from GenBank, dbEST, and RefSeq sequences clustered based on build 133 of the UniGene database and an additional 6500 transcripts identified from Unigene build 159. Scanned output files were visually inspected for hybridization artifacts and then analyzed by using robust multi-array average analysis (RMA). RMA is a well-established procedure that uses quantile normalization and a model-based signal calculation for determination of expression values in probe based microarray gene expression.[12] RMA is used to compute expression values for a set of Affymetrix microarray chips within an experiment. This process is robust to experimental noise and conditions and has been shown to reduce variability in expression calculations. RMA consists of three distinct steps: background adjustment, quantile normalization and summarization by median polish. The background adjustment step estimates and subtracts background levels from the perfect-match probes. This is followed by quantile normalization that equalizes chip intensity histograms to facilitate future cross-chip analysis. Finally, the probe-level data is combined into a single summary expression using a median polish.[12–14]

## Statistical Methods

### Sample size calculation

Following the approach proposed in *Tibshirani*, *et al*, we used the SAM software to generate an estimate of power.[15] We utilized PBMC data run on the same platform (Affymetrix HG-U133Plus 2.0) from liver transplant patients in *Martinez-Llordella*, *et al* to generate estimates for sample size and power.[5] This experimental data was utilized for power calculation, as no such data was available at time of study design from tolerant and non-tolerant HCT recipients. The goal was to identify a minimum sample number threshold to identify differentially expressed genes at this initial discovery stage of this work. Using 10 TOL and 10 non-TOL liver transplant patients, and false discovery rate (FDR) of 10%, we estimated 99% power to detect an effect size of 1.5 for differentially expressed genes, assuming there are approximately 233 truly significant genes. Thus, a minimum sample size of 20 total subjects was required. However, to maximize our power to detect differences, we used the entire available patient sample set for discovery. Differences observed in this current study will inform future power calculations in a subsequent prospective study.

### Analysis methods—Differential gene expression

The Significance Analysis of Microarrays (SAM) technique of *Tusher*, *et al* was employed to identify differentially expressed genes between phenotypic groups.[16] SAM was utilized for the two group (TOL vs. non-TOL) comparison with 10% FDR, and ≥ 1.5 fold difference in mean expression values. To account for confounding by immune suppression (absent in TOL vs. present in non-TOL cases), we employed the following analyses: We first utilized SAM to identify differentially expressed genes between TOL and non-TOL groups. Second, we compared each group (i.e. TOL vs. control, and separately non-TOL vs. control) to the healthy control group using SAM. Shared genes (unidirectionally different in both TOL and non-TOL with reference to controls) were considered non-informative and filtered out, and thus unique gene lists that distinguished TOL and non-TOL from controls were developed. Finally, for each group of interest (TOL or non-TOL), we retained only those genes from the initial two-group comparison that also were identified as unique genes in each comparison to control. Thus, the final gene list for the TOL group were those that distinguished TOL from both non-TOL and control, and the final gene list for the non-TOL group contained those that distinguished non-TOL from both TOL and controls. Functional Ontology Enrichment (MetaCore by GeneGo) with 5% FDR filter was utilized to identify enriched canonical pathways and cellular process networks.

### Analysis methods—Cell lineage-specific gene expression

Gene set enrichment analysis (GSEA) was used to investigate enrichment of experimental data for cell lineage-specific (CD4+, CD8+, CD14+, CD19+, CD56+, CD66+) human gene expression.[17,18]

### Analysis methods—Classifier construction

Using these final TOL and non-TOL gene lists, a classifier was constructed using the leave-10%-out cross-validation method using R (R Foundation for Statistical Computing, Vienna, Austria). The stability of this classifier was tested across configurations including a range of 20–80 total probe sets, and each iteration of the classifier included 10-fold cross-validation. Diagnostic accuracy of the developed classifier is also demonstrated through a receiver operating characteristics (ROC) plot.

### Analysis methods—Confirmation of differential gene expression

Differential expression of selected genes (those highly discriminative in our cross-validation work—selected at least 9–10 times in 10-fold cross-validation)—as well as those selected based on association with top-ranked enriched biologic process networks) was confirmed using NanoString nCounter technology.[19] All of the tolerant, non-tolerant, and healthy control subjects represented in the primary microarray work were studied with this technology.

A custom NanoString nCounter Gene Expression (GX) CodeSet with probes representing 43 genes was developed and the sample was processed on the NanoString nCounter Analysis System according to the manufacturer’s protocol (NanoString Technologies, Seattle WA). Briefly, one hundred nanograms of RNA was hybridized to the reporter and capture probes in a thermal cycler for 16 hours at 65°C. Washing and cartridge immobilization was performed on the NanoCounter PrepStation, and the cartridge was scanned at 555 fields of view (FOV) on the nCounter Digital Analyzer. The resulting. RCC files containing raw counts were checked for quality in the NanoString nSolver Analysis Software v1.1, and then exported for normalization and analysis.

### Analysis methods—Secondary matched-pair analysis

As a secondary analysis approach, a paired (matched TOL vs. non-TOL pairs) analysis utilizing Affymetrix MAS 5.0 comparison analysis for matched samples was performed. We also investigated shared differentially expressed genes between our data (TOL vs. non-TOL comparison) and previously published differential gene expression data following solid organ transplantation (TOL vs. non-tolerant comparator), and mapped shared genes to enriched pathways.[2–5]

## Results

### Patient characteristics

A total of 15 tolerant patients after HCT were identified and had sample collection. Two additional tolerant cases were identified, but were not able to participate in the study. A total of 17 non-tolerant comparators were selected based on age, time from HCT, and other clinical transplantation characteristics, and had samples collected. Demographic, transplantation, and GVHD characteristics of the included patients are detailed in Table 1. Finally, a total of 10 healthy volunteer control subjects were recruited. These were without acute or chronic illness, and were not on any medications. Median age of controls was 32.5 (range 27–59) years, and included 7 females and 3 males. The TOL and non-TOL patients did not significantly differ according to demographic, disease, or transplantation characteristics (Table 1). Note that Table 1 summarizes historical maximum GVHD severity and therapy, not GVHD activity or IS therapy at time of sample acquisition (i.e. tolerant patients by definition had no GVHD and were off IS at time of sample collection). These were adult patients with hematologic malignancies and disorders predominantly treated with myeloablative chemotherapy-based conditioning. The majority received peripheral blood stem cells from either matched sibling or matched unrelated doors. Initial GVHD prophylaxis was a calcineurin inhibitor together with either methotrexate or mycophenolate mofetil, and acute GVHD severity and treatment did not differ between groups.

**Table 1:** Comparison of patient, transplantation, and GVHD variables across tolerant and non-tolerant groups.

**Variable**	**Tolerant**	**Non-Tolerant**	**p value**
**Median age**	50	49	0.79
**Donor age**	38	52	0.11
**Condition**			
*AA*	1	0	0.39
*ALL*	2	3	
*AML*	3	7	
*CML*	0	1	
*FL*	2	2	
*HD*	0	1	
*IMF*	0	1	
*MCL*	2	0	
*MCL*, *MDS*	1	0	
*MDS*	3	1	
*MM*	0	1	
*MPD*	1	0	
**Stem cell source**			
*PBSC*	15	16	0.34
*BM*	0	1	
**Donor relation**			
*MMUD*	1	0	0.51
*MRD*	10	11	
*MUD*	4	6	
**Donor:Recipient gender matching**			
*F/F*	3	6	0.36
*F/M*	2	5	
*M/F*	2	1	
*M/M*	8	5	
**HLA matching**			
*matched*	14	17	0.28
*mismatched*	1	0	
**CMV serostatus for recipient:donor**			
*neg/neg*	4	10	0.13
*neg/pos*	1	2	
*pos/neg*	5	1	
*pos/pos*	5	4	
**Conditioning**			
*Bu/Cy*	1	2	0.35
*Bu/Flu*	8	14	
*Bu/Flu/ATG*	1	0	
*Bu/Flu/R*	1	0	
*Cy/ATG*	1	0	
*Cy/BCNU/VP16*	1	0	
*Cy/TBI*	1	1	
*Flu/Cy/R*	1	0	
*Pento/Bu/R*	1	0	
**aGVHD prophylaxis (agent 1)**			
*CSA*	1	2	0.51
*CSA/TAC*	1	0	
*TAC*	13	15	
**aGVHD prophylaxis (agent 2)**			
*MMF*	6	6	0.78
*MTX*	9	11	
**Max grade aGVHD**			
*None*	4	2	0.29
*I*	4	1	
*II*	5	11	
*III*	1	2	
*IV*	1	1	
**aGVHD treatment (agent 1)**			
*None*	7	7	0.15
*MMF*	0	1	
*Pred < 1mg/kg*	1	0	
*Pred 1mg/kg*	4	9	
*Pred 2mg/kg*	3	0	
**aGVHD treatment (agent 2)**			
*None*	10	13	0.03
*MMF*	2	4	
*Rapa*	3	0	
**Max grade cGVHD**			
*None*	9	0	0.0001
*Mild*	6	5	
*Moderate*	0	8	
*Severe*	0	4	
**cGVHD treatment (agent 1)**			
*Prednisone*	1	3	0.13
*ECP*	0	1	
*MMF*	0	3	
*TAC*	0	1	
*Rapa*	0	1	
*None*	14	8	
**cGVHD treatment (agent 2)**			
*MMF*	0	2	0.001
*MTX*	0	1	
*Rapa*	0	3	
*TAC*	0	2	
*none*	15	9	
**cGVHD treatment (agent 3)**			
CSA	0	2	< 0.0001
Prednisone	0	1	
Rapa	0	1	
none	15	13	

*categorical data compared with Fisher’s exact test or Chi-square, continuous data utilized wilcoxon rank sum test

* AA—aplastic anemia; ALL—acute lymphoblastic leukemia; AML—acute myelogenous leukemia; CML—chronic myelogenous leukemia; FL—follicular lymphoma; HD—Hodgkin lymphoma; IMF—idiopathic myelofibrosis; MCL—mantle cell lymphoma; MDS—myelodysplastic syndrome; MM—multiple myeloma; MPD—myeloproliferative neoplasm; PBSC—peripheral blood stem cells; BM—bone marrow harvested stem cells; MMUD—mismatched unrelated donor; MRD—matched sibling donor; MUD—matched unrelated donor; HLA—human leukocyte antigen; CMV—cytomegalovirus; neg—negative; pos—positive; Bu—busulfan; Cy—cyclophosphamide; Flu—fludarabine; ATG—anti-thymocyte globulin; R—rituximab; BCNU—carmustine; VP16—etoposide; TBI—total body irradiation; pento—pentostatin; CSA—cyclosporine; TAC—tacrolimus; MMF—mycophenolate mofetil; MTX—methotrexate; aGVHD—acute graft vs. host disease; pred—prednisone; rapa—rapamycin (sirolimus); ECP—extra-corporeal photopheresis; cGVHD—chronic graft vs. host disease

The TOL and non-TOL groups did significantly differ in their history of chronic GVHD, as the non-TOL patients had greater NIH Consensus global severity of chronic GVHD and greater extent of therapy delivered for chronic GVHD: Among the TOL patients, 9 had no history of chronic GVHD, and 6 had a prior maximum mild chronic GVHD. Of these, only one required the addition of any systemic IS for chronic GVHD therapy. Among the TOL patients with any history of chronic GVHD, this was completely resolved at a median of 25.3 months (range 17.6–39.7) prior to the study sample acquisition. In contrast, the maximum global severity of chronic GVHD among the non-TOL patients was 1 (n = 5), 2 (n = 8), or 3 (n = 4). Chronic GVHD organ involvement included skin (n = 11), eye (n = 6), mouth (n = 6), GI (n = 5), liver (n = 8), lung (n = 2), and fascia/joints (n = 1). Therapy delivered included prednisone and additional systemic immune suppressive therapies, and none had discontinued all IS by time of study sample acquisition. We examined potential confounding from the type of IS agents among non-TOL patients at the time of sample acquisition: We performed SAM analysis according to the methods described above to discern differential gene expression based on presence/absence of each type of IS agent, and compared these differentially expressed genes against differentially expressed genes that segregated the TOL and non-TOL phenotypic groups. As no genes overlapped, we report that the observed differential gene expression is not likely explained by effects from the presence or type of IS.

The median time from HCT to study sample acquisition (TOL 38.5 vs. non-TOL 39.5 months) did not differ between groups, p = 0.97. The median time from complete IS discontinuation to study sample acquisition among TOL patients was 19.15 (range 7.1–68) months.

### Immune cell subsets

Immune cell subsets were identified through evaluation of cell surface markers (Table 2). There was a suggestion toward increased total CD8+ T cells, and specifically CD8 αβ T cells in the TOL group. However, based on our pre-specified significance level of 0.01 in the setting of multiple comparisons, we did not observe significant differences in any of the studied immune subsets between TOL and non-TOL groups.

**Table 2:** Comparison of immune cell subsets among TOL and non-TOL patients.

**Cell subset**	**Phenotype**	**TOL**	**Non-TOL**	**p value**
Total CD3+	CD3+	62.4	56.3	0.28
Total CD4+	CD4+	25.9	31.9	0.52
CD4+ CD25-	CD4+/CD25-	22.7	30.6	0.48
Total CD8+	CD8+	32.1	18.2	0.052
CD8αα (NK, DC, IEL)	CD8 αα+	1.85	1.4	0.27
CD8αβ (alpha-beta CD8)	CD8αβ+	18.6	6.8	0.03
Memory CD8	CD8+/CD127+	32.8	30	0.9
Effector CD8	CD8+/CD127-	67.2	69.9	0.9
Regulatory T cells (Treg)	CD4+/CD25+/CD127-	1.6	1.5	0.7
Treg/CD8 ratio	Treg/CD8+CD25+	1.1	0.9	0.82
Monocytes	CD14+	9.8	11.1	0.26
Total B cells	CD19+	12.9	8.1	0.1
Plasmacytoid DC	IL-3RA+/HLA-DR+	0.12	0.13	0.24
Monocytoid DC	CD11c+/HLA-DR+	0.23	0.57	0.27
NK cells	CD16+/CD56+	11.5	13.7	0.58
NKT	CD3+/CD16+/CD56+	0.05	0.025	0.16

*Numbers indicate proportion of examined PBMC with the identified phenotype.

*NK—natural killer cell; DC—dendritic cell; IEL—intra-epithelial lymphocyte; Treg—regulatory T cell; NKT—NKT cells; TOL—tolerant patients; non-TOL—non-tolerant patients

### Two-group (TOL vs. non-TOL) analysis

In the initial two-group comparison, SAM identified 231 probe sets over- and 412 under-expressed in the TOL vs. non-TOL group. Enriched process networks included those related to NK cells (NK cell cytotoxicity), phagocytosis and antigen presentation (phagocytosis, phagosome in antigen presentation), B cell signaling (BCR pathway), and lymphocyte differentiation and signaling (T helper cell differentiation, TCR signaling, protein C signaling, anti-apoptosis mediated via MAPK and JAK/STAT, lymphocyte proliferation, JAK-STAT pathway, and Th17-derived cytokines) (Table 3). Individual probe sets represented in enriched process networks are listed in (S1 File).

**Table 3:** Enriched cellular process networks from TOL vs. non-TOL comparison.

Networks	p value	Ratio(involved) / (total)	
Inflammation_NK cell cytotoxicity	1.553E-09	22	164
Immune response_Antigen presentation	2.233E-07	21	197
Inflammation_Neutrophil activation	5.064E-06	20	219
Reproduction_Feeding and Neurohormone signaling	1.082E-05	19	211
Immune response_Phagocytosis	2.234E-05	19	222
Chemotaxis	4.077E-05	14	137
Cell adhesion_Amyloid proteins	4.853E-05	17	195
Cell adhesion_Platelet aggregation	1.924E-04	14	158
Immune response_T helper cell differentiation	2.050E-04	13	140
Inflammation_Interferon signaling	3.337E-04	11	110
Cell adhesion_Leucocyte chemotaxis	8.981E-04	15	205
Inflammation_Histamine signaling	1.264E-03	15	212
Proliferation_Lymphocyte proliferation	3.036E-03	14	209
Inflammation_IL-2 signaling	3.175E-03	9	104
Signal Transduction_Cholecystokinin signaling	3.610E-03	9	106
Autophagy_Autophagy	5.004E-03	6	55
Inflammation_IgE signaling	6.168E-03	10	136
Inflammation_Jak-STAT Pathway	8.599E-03	12	188
Proteolysis_Proteolysis in cell cycle and apoptosis	1.048E-02	9	125
Immune response_Phagosome in antigen presentation	1.122E-02	14	243
Immune response_TCR signaling	1.248E-02	11	174
Inflammation_Protein C signaling	1.318E-02	8	108
Apoptosis_Anti-Apoptosis mediated by external signals via MAPK and JAK/STAT	1.517E-02	11	179
Development_Neurogenesis_Axonal guidance	1.655E-02	13	230
Blood coagulation	1.937E-02	7	94
Cell adhesion_Cell junctions	1.960E-02	10	162
Inflammation_Amphoterin signaling	2.145E-02	8	118
Proliferation_Positive regulation cell proliferation	2.753E-02	12	221
Development_Regulation of angiogenesis	2.926E-02	12	223
Cardiac development_Wnt_beta-catenin, Notch, VEGF, IP3 and integrin signaling	3.068E-02	9	150
Signal transduction_WNT signaling	3.355E-02	10	177
Development_Blood vessel morphogenesis	3.390E-02	12	228
Immune response_IL-5 signalling	3.825E-02	4	44
Cell adhesion_Glycoconjugates	4.654E-02	9	162

*Cellular process networks are ranked in descending order based on p value for magnitude of enrichment of experimental data to annotated networks using MetaCore by GeneGo software (limited to those with p < 0.05)

The secondary matched paired analysis identified 255 probe sets over- and 150 under-expressed in the TOL vs. non-TOL groups. Enriched process networks included TCR signaling (p = 1.1E-05), T helper cell differentiation (p = 0.00086), BCR pathway signaling (p = 0.0029), and NK cell cytotoxicity (p = 0.009). This secondary approach invoking matched-pair analysis was conducted to reduce potential heterogeneity from other clinical factors. While we note that the primary (group-wise SAM comparison) and secondary (pair-wise comparison using Affymetrix MAS 5.0 comparison analysis for matched samples) employed different statistical methods, we examined the concordance in findings across these methods. Of the total genes identified in the secondary approach, 41% of them were concordant with the main analysis (S6 File). Differentially expressed genes in our analysis were compared with those identified in published comparisons of tolerant vs. non-tolerant comparators in liver and kidney transplantation, and these were mapped to enriched cellular process networks (Table 4).

**Table 4:** Enriched cellular process networks shared between current experimental data and published tolerance-associated gene expression data in solid organ transplantation.

**Networks**	**p value(solid organ) / (HCT)**	**min(p value)**	**Ratio(involved) / (total)**	
Inflammation_NK cell cytotoxicity	6.239e-3 / 2.916e-9	2.916E-09	30	164
Chemotaxis	8.023e-8 / 5.866e-5	8.023E-08	29	137
Inflammation_Neutrophil activation	5.617e-3 / 1.389e-7	1.389E-07	33	219
Cell adhesion_Amyloid proteins	2.722e-1 / 3.292e-7	3.292E-07	26	195
Immune response_Phagocytosis	1.065e-3 / 7.266e-7	7.266E-07	36	222
Immune response_Phagosome in antigen presentation	1.905e-6 / 9.972e-4	1.905E-06	37	243
Inflammation_IL-4 signaling	3.185e-6 / 2.647e-1	3.185E-06	18	115
Immune response_Antigen presentation	1.967e-3 / 6.449e-6	6.449E-06	31	197
Reproduction_Feeding and Neurohormone signaling	4.162e-2 / 1.739e-5	1.739E-05	27	211
Immune response_BCR pathway	3.083e-5 / 2.211e-2	3.083E-05	25	137
Cell adhesion_Leucocyte chemotaxis	1.140e-3 / 4.091e-5	4.091E-05	30	205
Cell adhesion_Platelet-endothelium-leucocyte interactions	5.144e-5 / 1.536e-1	5.144E-05	25	174
Development_Neurogenesis_Axonal guidance	3.878e-3 / 5.783e-5	5.783E-05	34	230
Development_EMT_Regulation of epithelial-to-mesenchymal transition	6.085e-5 / 7.855e-2	6.085E-05	31	226
Inflammation_Histamine signaling	4.106e-3 / 6.375e-5	6.375E-05	30	212
Cell adhesion_Platelet aggregation	2.115e-1 / 7.520e-5	7.520E-05	21	158
Inflammation_Amphoterin signaling	1.004e-2 / 2.085e-4	2.085E-04	20	118
Inflammation_TREM1 signaling	2.183e-4 / 7.069e-2	2.183E-04	21	145
Immune response_T helper cell differentiation	6.623e-2 / 2.839e-4	2.839E-04	19	140
Cell adhesion_Cell junctions	3.689e-1 / 3.518e-4	3.518E-04	20	162
Inflammation_Protein C signaling	1.950e-1 / 3.778e-4	3.778E-04	14	108
Inflammation_Interferon signaling	5.743e-4 / 1.677e-3	5.743E-04	19	110
Cell cycle_G2-M	1.203e-3 / 9.983e-1	1.203E-03	18	206
Signal Transduction_Cholecystokinin signaling	8.818e-2 / 1.264e-3	1.264E-03	14	106
Signal Transduction_TGF-beta, GDF and Activin signaling	1.291e-3 / 3.185e-1	1.291E-03	18	154
Cell adhesion_Integrin-mediated cell-matrix adhesion	1.822e-3 / 1.957e-1	1.822E-03	25	214
Cell adhesion_Glycoconjugates	2.092e-3 / 3.444e-3	2.092E-03	24	162
Proliferation_Positive regulation cell proliferation	1.753e-1 / 2.640e-3	2.640E-03	23	221
Apoptosis_Anti-Apoptosis mediated by external signals via MAPK and JAK/STAT	5.893e-2 / 2.808e-3	2.808E-03	23	179
Development_Regulation of angiogenesis	2.819e-3 / 1.683e-2	2.819E-03	27	223
Proteolysis_ECM remodeling	3.399e-3 / 2.528e-1	3.399E-03	12	85
Development_Blood vessel morphogenesis	3.546e-3 / 4.204e-2	3.546E-03	26	228
Inflammation_IL-2 signaling	1.732e-1 / 3.956e-3	3.956E-03	13	104
Proliferation_Lymphocyte proliferation	3.424e-1 / 4.093e-3	4.093E-03	20	209
Signal transduction_ERBB-family signaling	5.399e-3 / 1.889e-1	5.399E-03	11	75
Autophagy_Autophagy	1.379e-2 / 5.872e-3	5.872E-03	9	55
Immune response_TCR signaling	2.335e-2 / 6.104e-3	6.104E-03	22	174
Cytoskeleton_Regulation of cytoskeleton rearrangement	6.295e-3 / 1.003e-1	6.295E-03	22	183
Apoptosis_Apoptotic mitochondria	6.331e-3 / 9.061e-1	6.331E-03	9	77
Proliferation_Negative regulation of cell proliferation	6.599e-3 / 1.899e-1	6.599E-03	20	184
Cytoskeleton_Actin filaments	1.040e-1 / 6.673e-3	6.673E-03	20	176
Inflammation_Kallikrein-kinin system	6.915e-3 / 1.937e-1	6.915E-03	21	185
Inflammation_IgE signaling	1.184e-1 / 7.734e-3	7.734E-03	17	136
Immune response_Th17-derived cytokines	6.344e-2 / 9.123e-3	9.123E-03	15	98
Development_Cartilage development	9.340e-3 / 3.195e-1	9.340E-03	9	66
Inflammation_Jak-STAT Pathway	3.921e-2 / 1.103e-2	1.103E-02	21	188
Proteolysis_Proteolysis in cell cycle and apoptosis	4.732e-1 / 1.283e-2	1.283E-02	13	125
Signal transduction_Leptin signaling	1.411e-2 / 2.143e-1	1.411E-02	13	106
Signal transduction_WNT signaling	1.899e-1 / 1.759e-2	1.759E-02	19	177
Apoptosis_Anti-apoptosis mediated by external signals via NF-kB	1.860e-2 / 1.173e-1	1.860E-02	14	111

*Cellular process networks are ranked in descending order based on p value for magnitude of enrichment to annotated networks using MetaCore by GeneGo software (for each process network, solid organ = published solid organ transplant data,[2–5] and HCT = HCT experimental data.

### Three group (TOL vs. non-TOL vs. control) analysis

SAM identified 643 probe sets differentially expressed between TOL and non-TOL groups. The TOL vs. control analysis identified 5,687 probe sets, of which 2,273 were unique after filtering out non-informative shared probe sets (those represented in both TOL vs. control and non-TOL vs. control lists and unidirectionally different from control). The non-TOL vs. control analysis identified 4,788 probe sets, of which 1,376 were unique. The final TOL list contained 281 probe sets, which were differentially expressed in the TOL group vs. both the non-TOL and control groups. The final non-TOL list contained 122 probe sets which were differentially expressed compared to both TOL and control groups.

**Fig 1:**
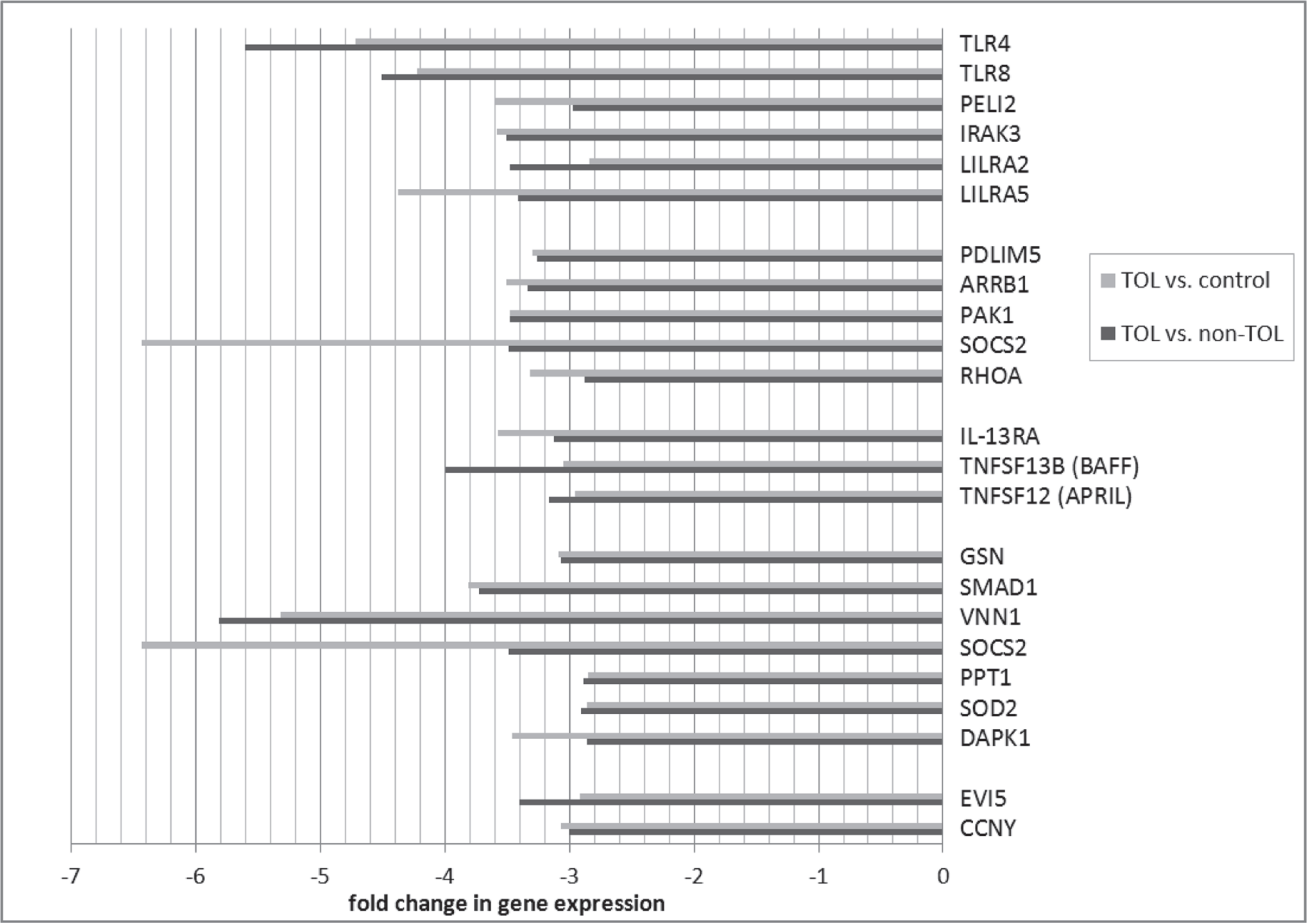
Direction and magnitude of change in TOL group vs. non-TOL and control: Genes with decreased expression in TOL group. *TLR4—toll-like receptor 4; TLR8—toll-like receptor 8; PELI2—pellino homolog 2; IRAK3—interleukin-1 receptor-associated kinase 3; LILRA2—leukocyte immunoglobulin-like receptor, subfamily A (with TM domain), member 2; LILRA5—leukocyte immunoglobulin-like receptor, subfamily A (with TM domain), member 5; PDLIM5—PDZ and LIM domain 5; ARRB1—arrestin, beta 1; PAK1—p21 protein (Cdc42/Rac)-activated kinase 1; SOCS2—suppressor of cytokine signaling 2; RHOA—ras homolog gene family, member A; IL-13RA—interleukin 13 receptor, alpha 1; TNFSF13B—tumor necrosis factor (ligand) superfamily, member 13b (BAFF); TNFSF12—tumor necrosis factor (ligand) superfamily, member 12 (APRIL); GSN—gelsolin; SMAD1—SMAD family member 1; VNN1—vanin 1; PPT1—palmitoyl-protein thioesterase 1; SOD2—superoxide dismutase 2, mitochondrial; DAPK1—death-associated protein kinase 1; EVI5—ecotropic viral integration site 5; CCNY—cyclin Y.

**Fig 2:**
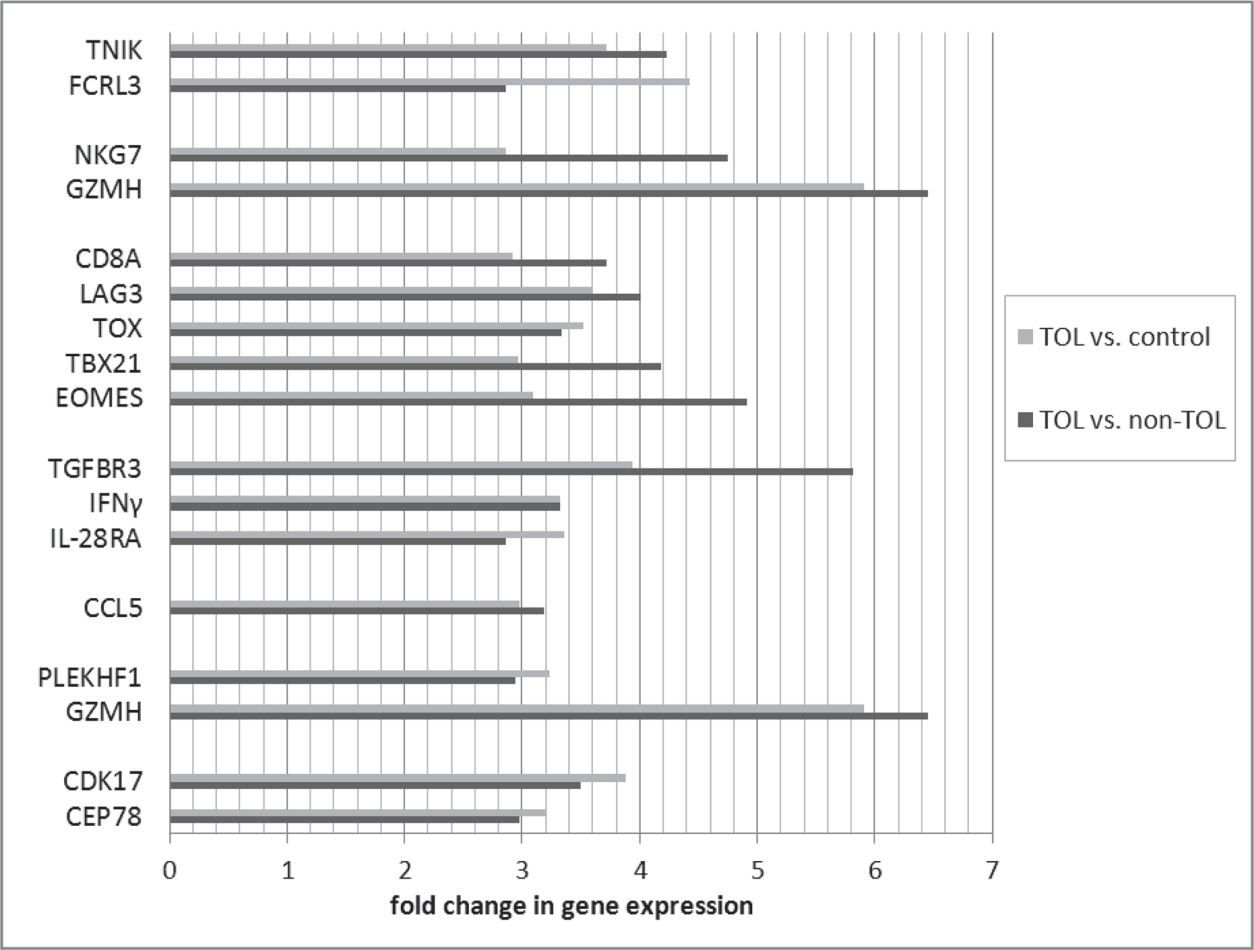
Direction and magnitude of change in TOL group vs. non-TOL and control: Genes with increased expression in TOL group. *TNIK—TRAF2 and NCK interacting kinase; FCRL3—Fc receptor-like 3; NKG7—natural killer cell group 7 sequence; GZMH—granzyme H (cathepsin G-like 2, protein h-CCPX); CD8A—CD8a molecule; LAG3—lymphocyte-activation gene 3; TOX—thymocyte selection-associated high mobility group box; TBX21—T-box 21 (T-bet); EOMES—eomesodermin; TGFBR3—transforming growth factor, beta receptor III; IFNγ—interferon, gamma; IL-28RA—interleukin 28 receptor, alpha (interferon, lambda receptor); CCL5—chemokine (C-C motif) ligand 5; PLEKHF1—pleckstrin homology domain containing, family F (with FYVE domain) member 1; GZMH—granzyme H (cathepsin G-like 2, protein h-CCPX); CDK17—cyclin-dependent kinase 17; CEP78—centrosomal protein 78kDa.

**Fig 3:**
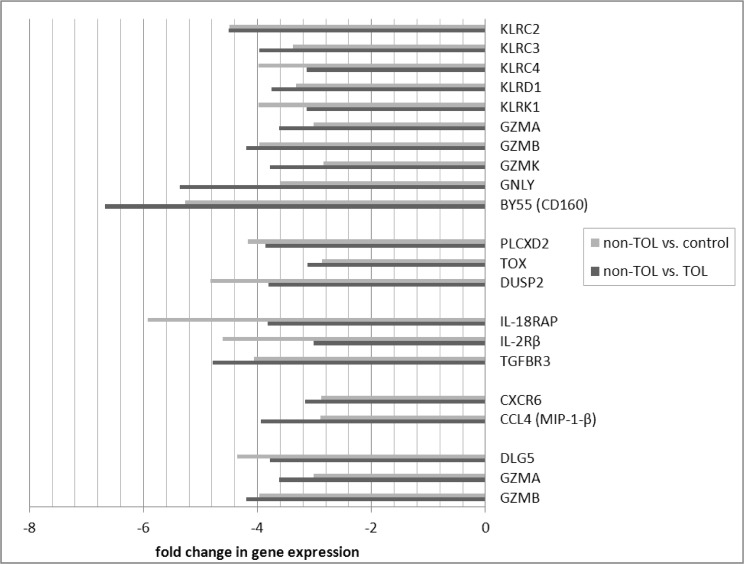
Direction and magnitude of change in non-TOL group vs. TOL and control: Genes with decreased expression in non-TOL group. *KLRC2—killer cell lectin-like receptor subfamily C, member 2 (NKG2C); KLRC3—killer cell lectin-like receptor subfamily C, member 3 (NKG2E); KLRC4—killer cell lectin-like receptor subfamily C, member 4 (NKG2F); KLRD1—killer cell lectin-like receptor subfamily D, member 1 (NKG2A); KLRK1—killer cell lectin-like receptor subfamily K, member 1 (NKG2D); GZMA—granzyme A (granzyme 1, cytotoxic T-lymphocyte-associated serine esterase 3); GZMB—granzyme B (granzyme 2, cytotoxic T-lymphocyte-associated serine esterase 1); GZMK—granzyme K (granzyme 3; tryptase II); GNLY—granulysin; BY55 (CD160)—CD160 molecule; PLCXD2—phosphatidylinositol-specific phospholipase C, X domain containing 2; TOX—thymocyte selection-associated high mobility group box; DUSP2—dual specificity phosphatase 2; IL-18RAP—interleukin 18 receptor accessory protein; IL-2Rβ—interleukin 2 receptor, beta; TGFBR3—transforming growth factor, beta receptor III; CXCR6—chemokine (C-X-C motif) receptor 6; CCL4 (MIP-1-β)—chemokine (C-C motif) ligand 4; DLG5—discs, large homolog 5; GZMA—granzyme A (granzyme 1, cytotoxic T-lymphocyte-associated serine esterase 3); GZMB—granzyme B (granzyme 2, cytotoxic T-lymphocyte-associated serine esterase 1).

**Fig 4:**
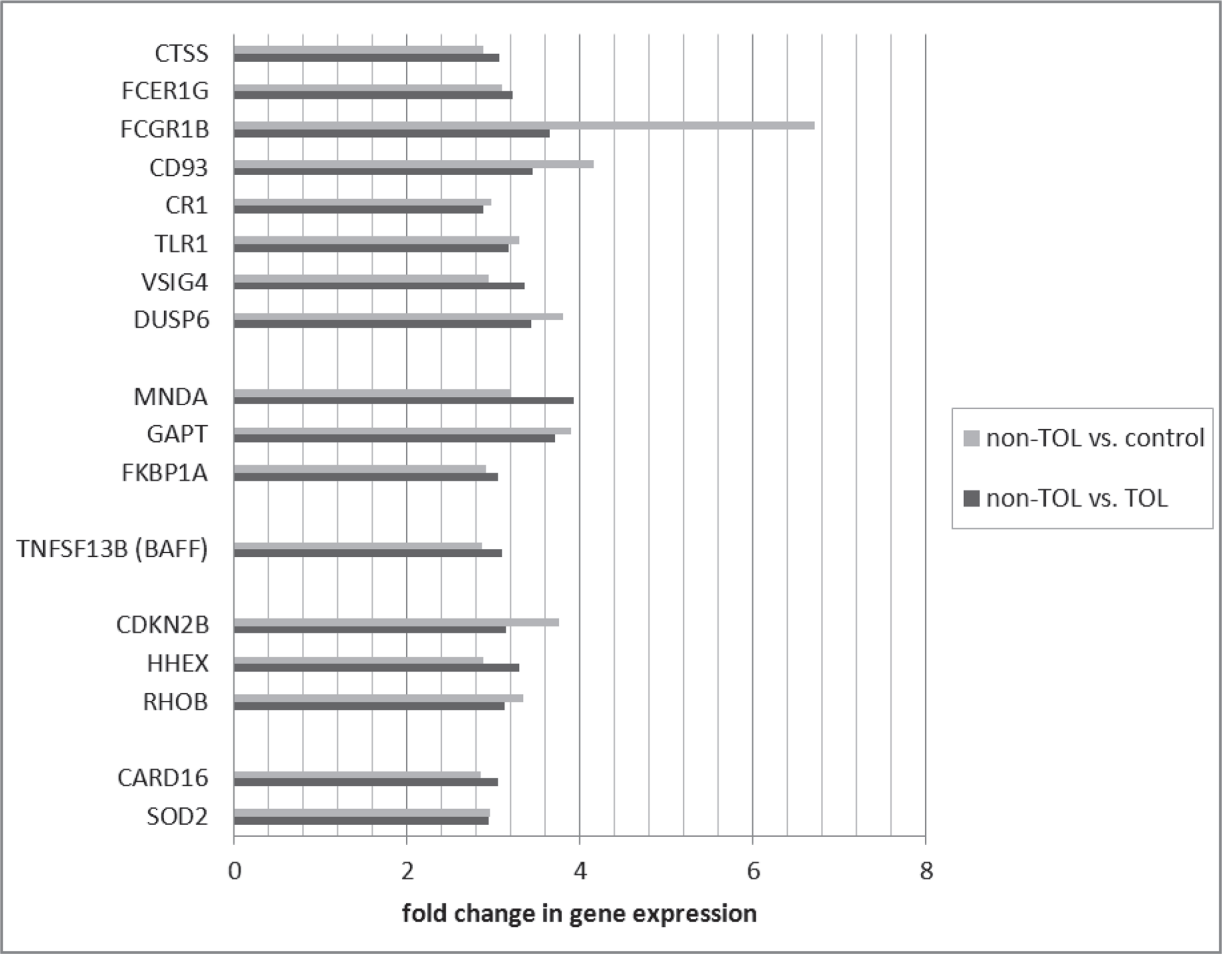
Direction and magnitude of change in non-TOL group vs. TOL and control: Genes with increased expression in non-TOL group. *CTSS—cathepsin S; FCER1G—Fc fragment of IgE, high affinity I, receptor for; gamma polypeptide; FCGR1B—Fc fragment of IgG, high affinity Ib, receptor (CD64); CD93—CD93 molecule; CR1—complement component (3b/4b) receptor 1; TLR1—toll-like receptor 1; VSIG4—V-set and immunoglobulin domain containing 4; DUSP6—dual specificity phosphatase 6; MNDA—myeloid cell nuclear differentiation antigen; GAPT—GRB2-binding adaptor protein, transmembrane; FKBP1A—FK506 binding protein 1A, 12kDa; TNFSF13B—tumor necrosis factor (ligand) superfamily, member 13b (BAFF); CDKN2B—cyclin-dependent kinase inhibitor 2B (p15, inhibits CDK4); HHEX—hematopoietically expressed homeobox; RHOB—ras homolog gene family, member B; CARD16—caspase recruitment domain family, member 16; SOD2—superoxide dismutase 2, mitochondrial.

Differentially expressed probe sets in the TOL and non-TOL groups were enriched for immune response genes focused in the innate immune response, NK cytotoxicity, lymphocyte signaling and regulation, apoptosis and cell cycle control. The direction and magnitude of differences with respect to each comparison group is represented in Figs. 1–4 for selected genes; from the total 281 TOL and 122 non-TOL probe sets, these genes were selected for presentation based on their association with top-scored cellular process networks, > 2-fold change vs. comparator groups, and relevance to established mechanisms of immune tolerance.

### Classifier construction and cross-validation

**Fig 5:**
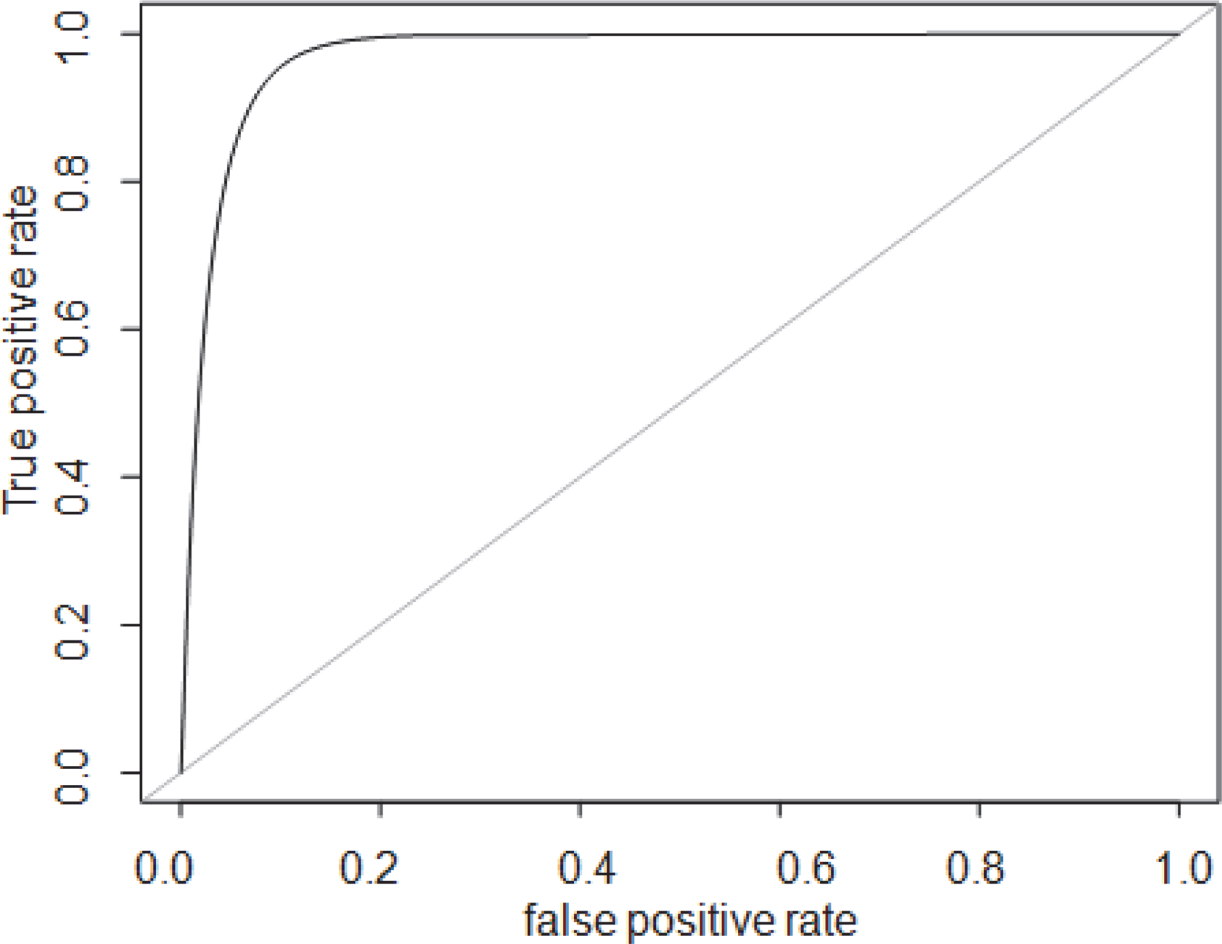
Receiver operating characteristic (ROC) plot for diagnostic accuracy of gene classifier. *ROC plot for diagnostic accuracy presents true positive rate vs. false positive rate (or sensitivity x 1-specificity) for gene expression-based phenotypic classifier of TOL and non-TOL patient groups. AUC 0.97 (95% CI 0.82–0.97).

**Fig 6:**
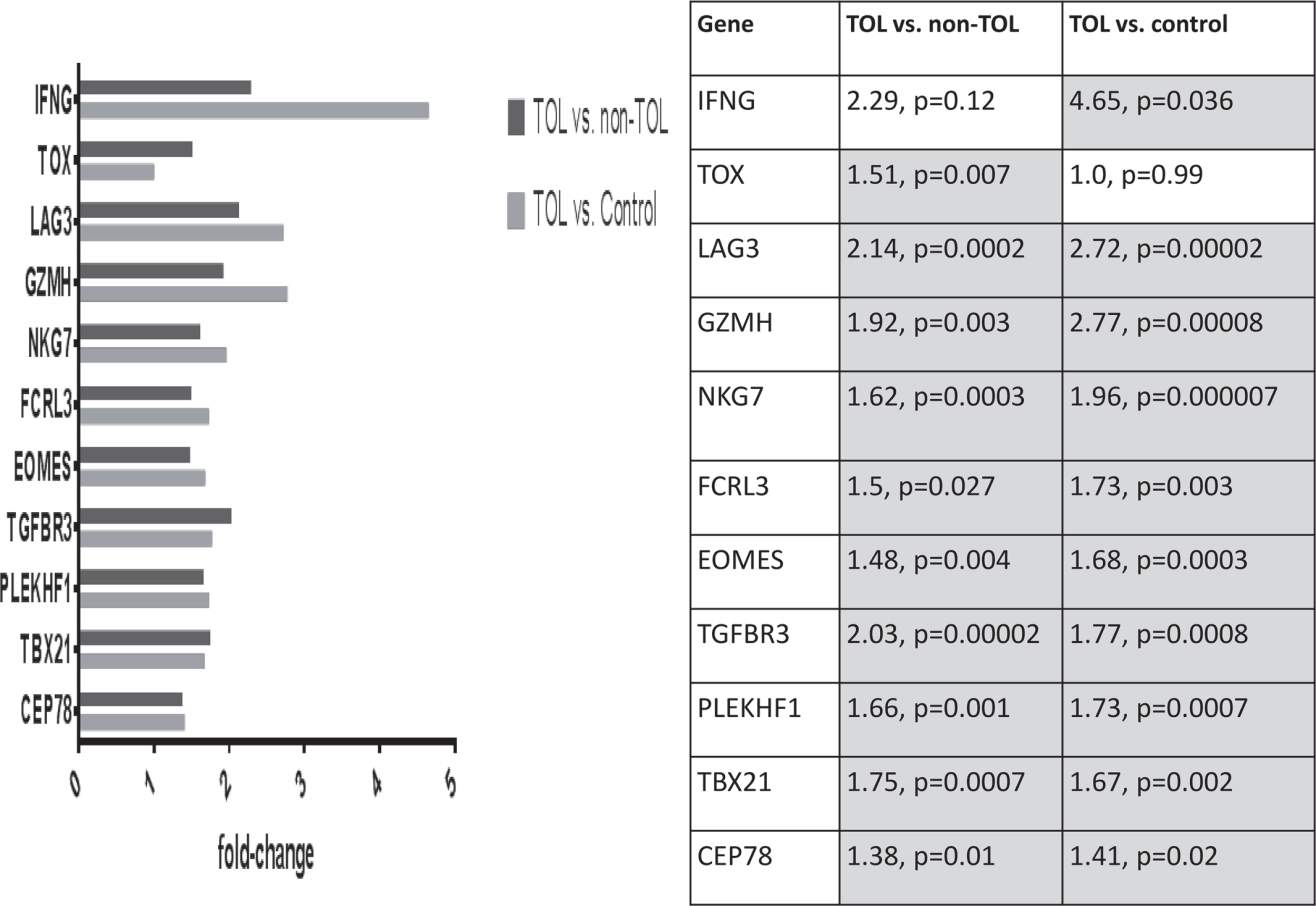
Confirmation of differential gene expression using NanoString: Genes with increased expression in TOL group.

**Fig 7:**
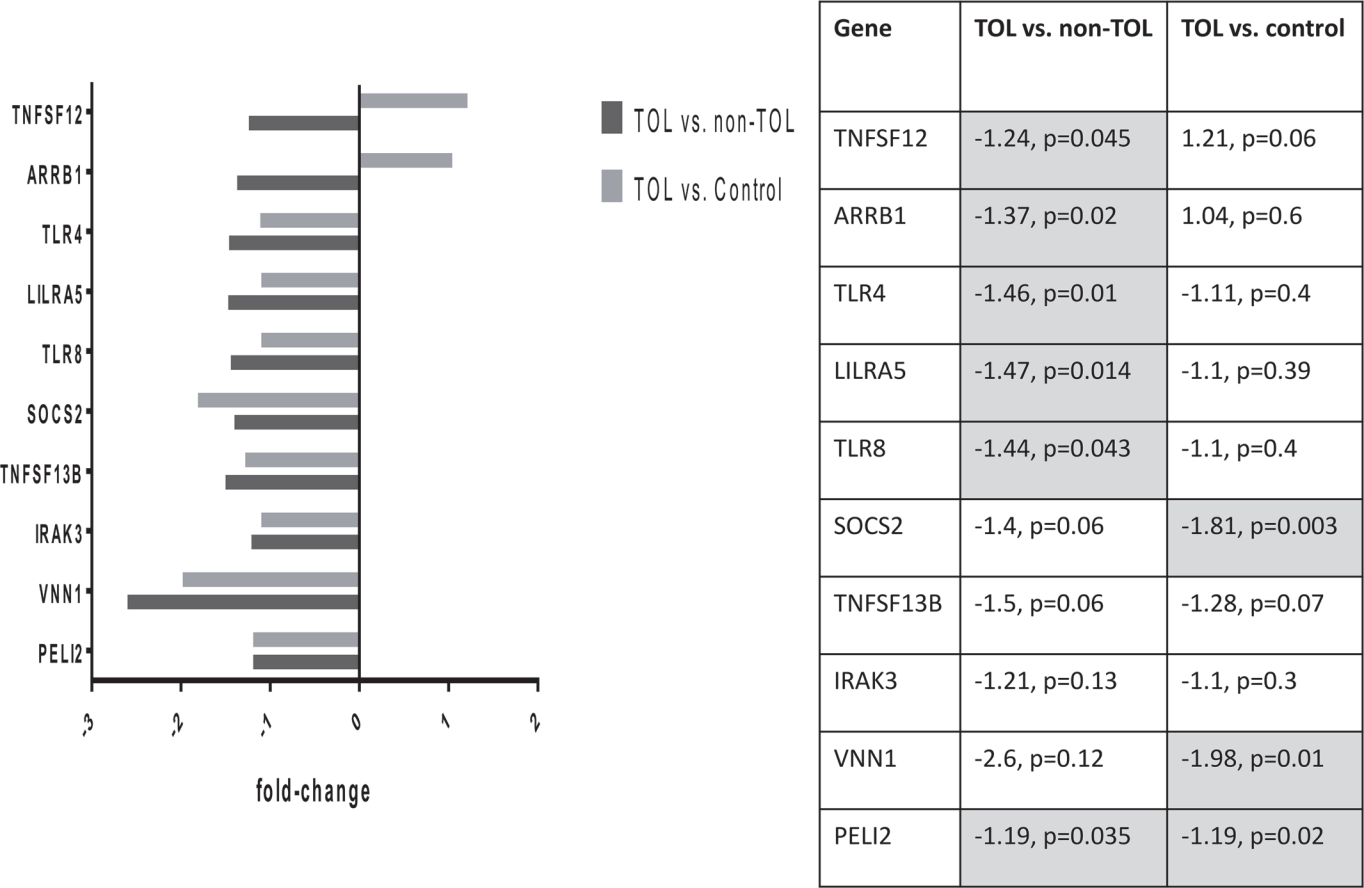
Confirmation of differential gene expression using NanoString: Genes with decreased expression in TOL group.

**Fig 8:**
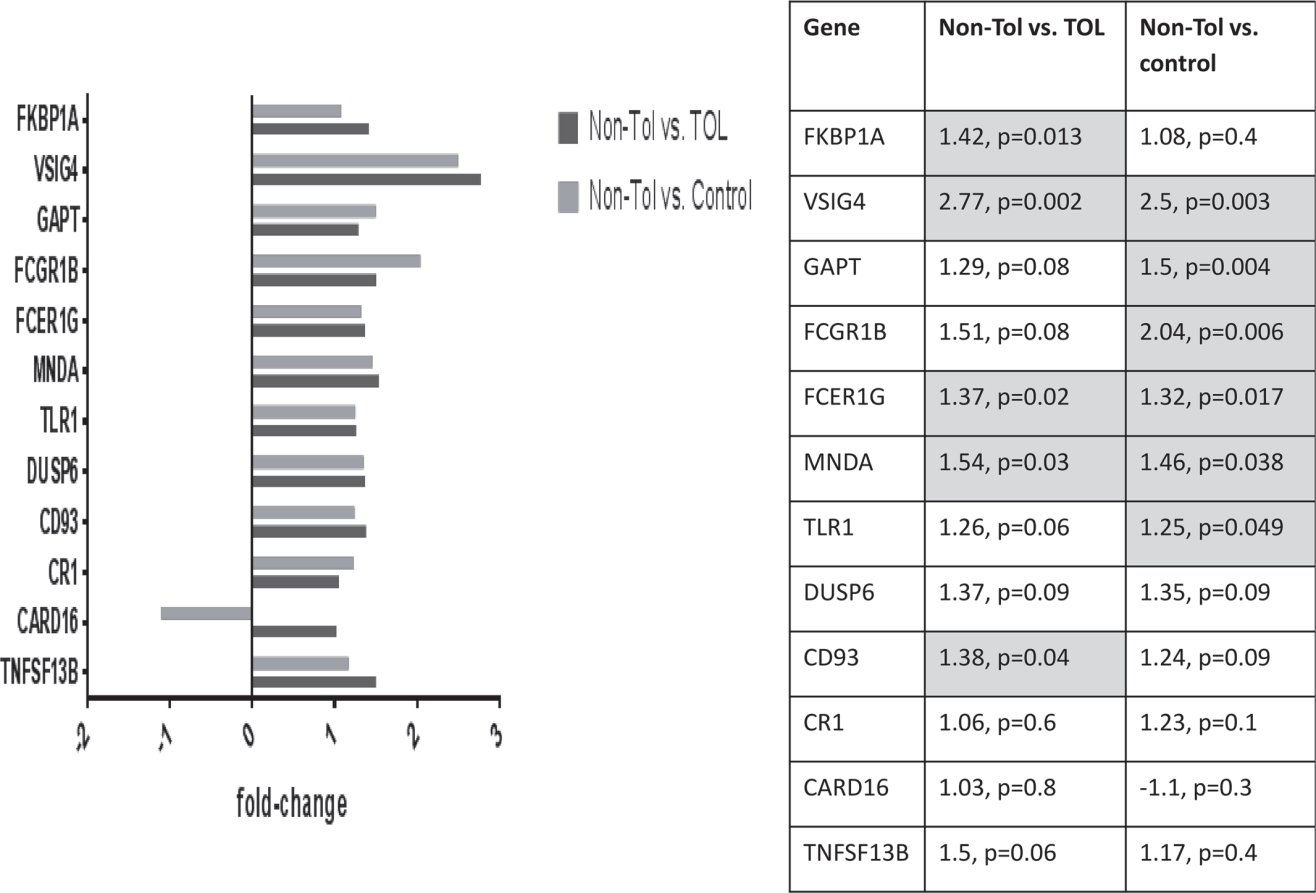
Confirmation of differential gene expression using NanoString: Genes with increased expression in non-TOL group.

**Fig 9:**
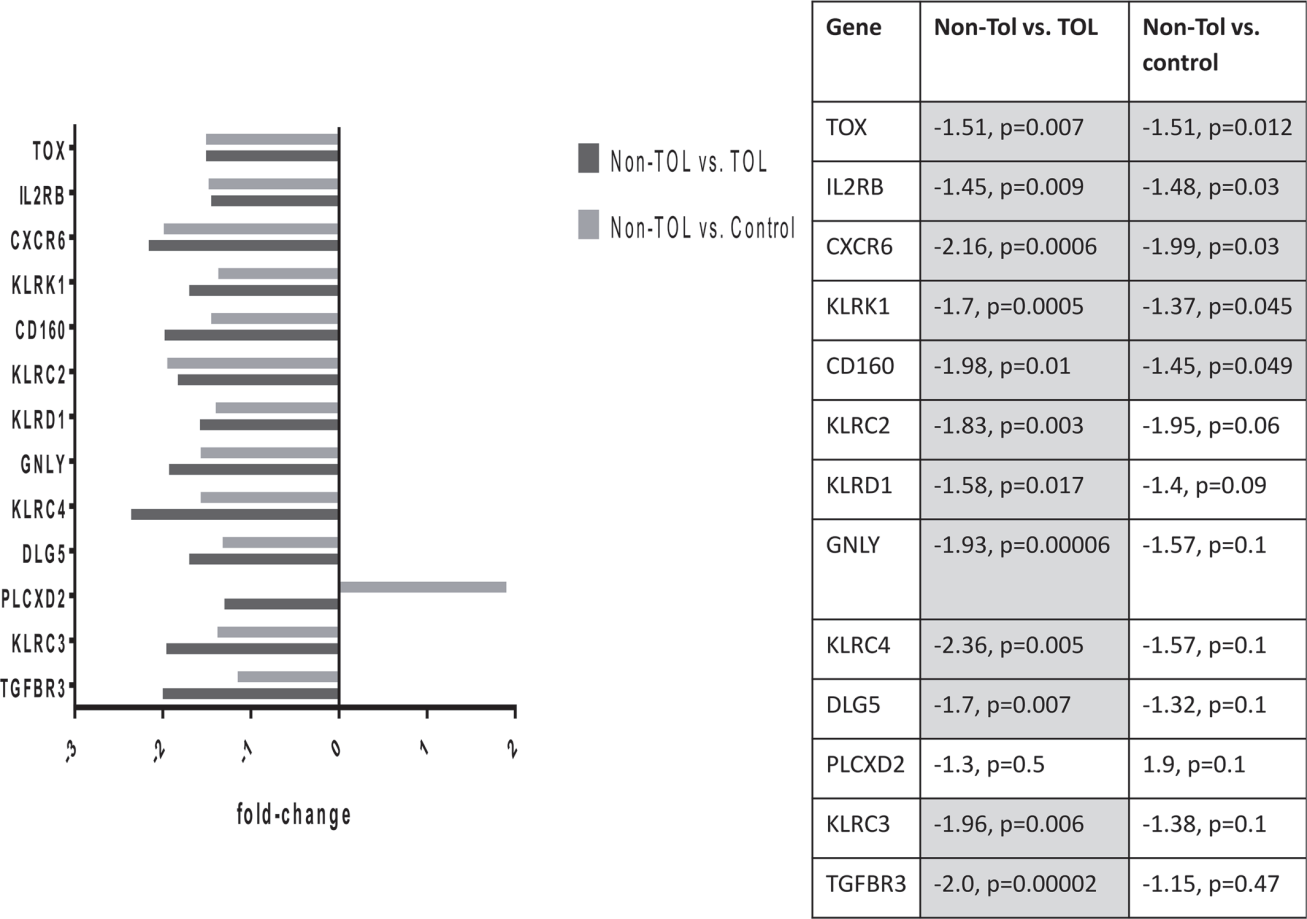
Confirmation of differential gene expression using NanoString: Genes with decreased expression in non-TOL group.

The leave-k-out cross-validation method was utilized to train a classifier for the phenotypic groups (TOL vs. non-TOL) based on the observed differential gene expression. For each of 10 rounds of cross-validation, 10% of the total sample was left out for testing the classifier. A number equal to 10% of the total samples was obtained by randomly selecting members from each of the two groups in order to have a balanced hold-out set. This was repeated 10 times with unique samples selected at each cross validation. For each validation the remaining 90% of samples where used to select the features and train the artificial neural network (ANN). The number of correct and incorrect classifications were computed for each cross-validation and detailed in a confusion matrix. The overall weighted accuracy was determined for the classifier. An accurate classifier (> 90% accuracy, correctly classifying 14/15 TOL cases and 15/17 non-TOL cases) was developed only utilizing 20 probe sets, and classifier accuracy was stable (ranging from 87.5 to 90.6%) across the range of included (20–80 total) probe sets. The highest ranked (selected for classifier development 9–10 times out of 10 total rounds of cross-validation) probe sets and corresponding genes from the 20-probeset classifier are listed in Table 5, and ROC diagnostic accuracy plot is shown in Fig. 5. Full data on cross-validation results are presented as S2 and S3 Files. Differential gene expression confirmed by NanoString is shown in Figs. 6–9.

**Table 5:** Top probe sets and corresponding genes selected in classifier construction and leave-10%-out cross-validation.

**Number of times selected**	**Probe set ID**	**Gene symbol**	**Gene name**
10	235230_at	PLCXD2	phosphatidylinositol-specific phospholipase C, X domain containing 2
10	231776_at	EOMES	eomesodermin
10	226625_at	TGFBR3	transforming growth factor, beta receptor III
10	219566_at	PLEKHF1	pleckstrin homology domain containing, family F (with FYVE domain) member 1
10	214119_s_at	FKBP1A	FK506 binding protein 1A, 12kDa
10	206974_at	CXCR6	chemokine (C-X-C motif) receptor 6
10	206486_at	LAG3	lymphocyte-activation gene 3
10	204787_at	VSIG4	V-set and immunoglobulin domain containing 4
10	204731_at	TGFBR3	transforming growth factor, beta receptor III
10	204530_s_at	TOX	thymocyte selection-associated high mobility group box
10	1557985_s_at	CEP78	centrosomal protein 78kDa
9	218832_x_at	ARRB1	arrestin, beta 1

### Cell lineage-specific gene expression enrichment

The GSEA method was used to determine enrichment for cell lineage-specific gene expression separately for the final TOL and non-TOL gene lists (Table 6) and differential gene expression discerned from the initial 2-group SAM comparison of TOL and non-TOL subjects (Table 7). Additional supplemental data is provided (S4 File).

**Table 6:** Cell lineage enrichment analysis conducted using final TOL and non-TOL gene lists.

	**Cell lineage**	**Size**	**ES**	**NES**	**P value**	**FDR**
**Final TOL gene list**						
	CD56	5	0.84	2.26	< 0.0001	< 0.0001
**Final non-TOL gene list**						
	CD66	9	0.71	2.3	< 0.0001	< 0.0001
	CD56	13	−0.59	−2.3	< 0.0001	0.001

*TOL—tolerant; non-TOL—not tolerant; ES—enrichment score; NES—normalized enrichment score; p value—significance level; FDR—false discovery rate.

**Table 7:** Cell lineage enrichment analysis conducted using Initial 2 group SAM comparison (TOL vs. non-TOL).

**Cell lineage**	**Size**	**ES**	**NES**	**P value**	**FDR**
CD14	41	−0.47	−3.24	< 0.0001	< 0.0001
CD56	21	0.71	3.76	< 0.0001	< 0.0001
CD66	47	−0.45	−3.24	< 0.0001	< 0.0001

*Data are presented for cell-lineage specific gene sets for which significant enrichment was demonstrated. Data for CD4, CD8, and CD19 not presented (p = NS).

*TOL—tolerant; non-TOL—not tolerant; ES—enrichment score; NES—normalized enrichment score; p value—significance level; FDR—false discovery rate.

### Replication in an independent patient set

Comprehensive search for comparable independent patient sets was performed using GEO Datasets. We identified one comparable study (Agilent microarray, 35 cGVHD and 28 non-GVHD samples).[20] While important differences limited this comparison (patient characteristics, time post-HCT, institution, time period, microarray platform), we attempted to replicate our findings in this comparable data set. We applied SAM analysis (1.5 fold change in gene expression, FDR of 5%) to this data set, and identified 36 probe sets that overlapped between this and our primary data set. These differentially expressed genes were mapped to annotated pathways using GeneGo by MetaCore (S5 File).

## Discussion

Current management of IS after HCT is empiric and complicated by risk of GVHD and its associated morbidity and mortality. Biologic markers of immune tolerance after HCT may provide mechanistic insight into transplantation tolerance and guide IS treatment after HCT. We studied differential gene expression in the peripheral blood of TOL and non-TOL HCT recipients, and healthy controls. The principle finding from our analysis is that differential gene expression in PBMC can provide insight into transplantation tolerance biology, and can be utilized to develop a genomic classifier with a high degree of accuracy to distinguish TOL from non-TOL patients after HCT.

Differentially expressed genes in the TOL group were enriched for immune response pathways and recapitulated experimental mechanisms of immune tolerance: Expression of leukocyte immunoglobulin-like receptors (LILRA5, LILRA2) was decreased in the TOL group; these LILR are activating and associated with release of pro-inflammatory cytokines.[21] The Ig receptor superfamily member FCRL3 was over-expressed in the TOL group; this molecule is involved in immune regulation, negatively regulates B cell receptor signaling,[22] and may distinguish a distinct subset of Treg.[23] Major components of the toll-like receptor signaling cascade (TLR4, TLR8, PELI2, IRAK3) were under-expressed as well; TLR/MyD88 signaling plays a key role in experimental models of transplantation tolerance,[24,25] and TLR4 inactivation protects against GVHD.[26] Several important cell signaling molecules were over-expressed: TOX is known to be involved in CD4 T cell lineage development, and important for Treg and CD1d-dependent NKT cells.[27] LAG3, a major negative regulator of CD4 and CD8 T cell activation and important for Treg homeostasis, function, and inhibition of DC activation, was over-expressed.[28–30] Conversely, SOCS2 (involved in DC maturation),[31] and beta arrestin 1 (involved in T cell activation, enhances transcription of IFN-γ and IL-17; increased in primary biliary cirrhosis patients)[32,33] were decreased. Among cytokines and their receptors, TOL patients had decreased expression of IL-13RA (IL-13 induces B cell proliferation and differentiation, and is expressed on Th17 cells), as well as BAFF and APRIL (major B cell activating TNF ligand family members implicated in human chronic GVHD).[34–36] Conversely, TGFBR3 (TGF-β co-receptor relevant to TGF-β receptor complex stability and signaling),[37] expression was increased. This finding is in keeping with the established relevance of TGF-β in immune tolerance, and previous seminal work demonstrating the importance of TGF-β pathway mediators in human GVHD.[38] As well, IFN-γ was increased in TOL patients. This has been demonstrated to have both pro-inflammatory and immuno-regulatory actions,[39] importance in migration of Treg and conventional T cells to GVHD target organs,[40] and to mediate immune-regulatory function in FoxP3+ Tregs in experimental GVHD.[41] In keeping with published data in solid organ transplantation tolerance, TOL patients had decreased expression of anti-apoptotic (DAPK1, SOD2, PPT1, SOCS2, VNN1, SMAD1, GSN), and increased expression of pro-apoptotic (GZMH, PLEKHF1) mediators, as well as involvement of cell cycle control genes.

Differentially expressed genes in the non-TOL group were strongly associated with NK cell cytotoxicity, antigen presentation, lymphocyte proliferation, and cell cycle and apoptosis cellular process networks: Multiple NK cell/lectin receptors (By55/CD160, KLRK1, KLRD1, KLRC4, KLRC3, KLRC2) and cytolytic effectors (granulysin, and granzymes A, B, and K) were under-expressed in the non-TOL group with respect to both TOL and control subjects.[42] Tolerogenic activity of NK cells has been related to killing of activated T cells, production of IL-10, competition with CD8+ T effectors for IL-15, and killing of antigen-presenting DC.[1] While we did not detect decrease in absolute NK cell numbers, these gene expression findings are in keeping with a cohesive finding of NK deficiency in human chronic GVHD,[43,44] as well as the primacy of NK-associated gene expression changes (including specifically CD160 and NKG7) in distinguishing tolerant vs. non-tolerant liver transplant recipients in *Martinez-Llordella, et al.[5]* There was over-expression of TLR/MyD88 signaling (DUSP6, TLR1), complement receptors (VSIG4, CR1, CD93), and Fc receptors (FCGR1B, FCER1G), again highlighting the important role of the innate immune system. Among signaling mediators, GAPT (GRB2-binding adaptor protein associated with B cell activation),[45] and MNDA (myeloid cell nuclear differentiation antigen expressed in cells of the granulocyte-monocyte lineage and involved in response to interferon) were increased; interferon-inducible Ifi200-family genes (including MNDA) have been associated with autoimmune disorders, including systemic lupus erythematosis.[46,47] In contrast to TOL, the non-TOL patients had increased BAFF, and decreased TGFBR3. In keeping with findings after solid organ transplantation, non-TOL patients had increased expression of anti-apoptotic (SOD2, CARD16) and decreased expression of pro-apoptotic (GZMB, GZMA, DLG5) mediators, and involvement of molecules relevant to cell cycle control.

Our analysis is strengthened by the following: First, TOL and non-TOL patients were precisely matched to mitigate confounding by other clinical characteristics. As well, we invoked healthy controls, and filtered out non-informative genes to account for confounding due to the absence or presence of immune suppressive agents. In addition, all post-HCT patient and control subject samples were freshly obtained, and processed identically in a single center to minimize technical artifacts. Finally, we also considered the impact of sample cellular composition, however we did not detect significant differences in immune subsets across TOL and non-TOL groups. Our cell lineage-specific gene enrichment analyses, however, provide hypotheses for subsequent investigation.

We note the following limitations: First, candidates identified in this single sample cross-sectional study require further validation in a larger independent cohort; such validation was attempted using a previously reported independent cohort,[20] but was unsuccessful. As well, despite rigorous efforts to account for confounding effects, it is not known whether the developed classifier in this current approach will perform comparably in a prospective study in which samples are drawn prior to IS discontinuation and subsequent clinical phenotype is observed. Multiple time-point prospective trials may also be able to detect dynamic changes over time associated with development of immune tolerance. Second, there are several considerations in the clinical samples obtained: While tolerance associated changes in gene expression in PBMC may not reflect those changes occurring in other compartments, it represents a clinically feasible approach. Similarly, study of flow-sorted individual immune cell populations is of interest, but limited by its time- and resource-intense demands. Next, our study was limited to transcriptional analysis by microarray. Additional insight may be gained with RNA sequencing, proteomics, and other technologies. Next, we acknowledge that the patient, disease, and transplantation characteristics of our study sample are not representative of the overall diversity of these factors among the overall HCT population; for example, there is limited representation here of alternative donors (mismatched unrelated, umbilical cord blood, or haploidentical). These and other limitations speak to the need for larger studies representative of this diversity. Finally, while comparison of post-HCT tolerance-associated gene expression to that reported after solid organ transplantation is of interest, there are several differences (e.g. type of non-tolerant comparator group, timing of sample acquisition post-transplant, additional clinical confounding variables, variation in platform and analytic methods utilized) that limit this comparison. Thus, synthesis of immune tolerance associated gene expression changes across these studies based on current evidence is limited.

In summary, we conclude that differential gene expression from peripheral blood samples can be utilized to accurately classify TOL and non-TOL patients post-HCT. If validated, the clinical application of this technology could facilitate personalized discontinuation of IS after HCT.

## Supporting Information

S1 File Individual genes represented in top enriched pathways for TOL vs. non-TOL comparison. (XLS)

S2 File Complete results of leave-10%-out cross-validation method, including individual gene lists and accuracy. (XLSX)

S3 File Complete results of leave-10%-out cross-validation method, including individual gene lists and accuracy. (DOCX)

S4 File Detail on cell lineage-specific gene expression identified through GSEA analysis. (XLSX)

S5 File Top-ranked enriched pathways and associated individual differentially expressed genes identified in external replication cohort analysis. (XLS)

S6 File Genes from secondary paired analysis that were shared with differentially expressed genes from the primary SAM two-group analysis. (XLS)

## References

[1] Bluestone JA (2011) Mechanisms of tolerance. Immunol Rev 241: 5–19. 10.1111/j.1600-065X.2011.01019.x 21488886

[2] Brouard S, Mansfield E, Braud C, Li L, Giral M, et al (2007) Identification of a peripheral blood transcriptional biomarker panel associated with operational renal allograft tolerance. Proc Natl Acad Sci U S A 104: 15448–15453. 17873064 10.1073/pnas.0705834104 PMC2000539

[3] Braud C, Baeten D, Giral M, Pallier A, Ashton-Chess J, et al (2008) Immunosuppressive drug-free operational immune tolerance in human kidney transplant recipients: Part I. Blood gene expression statistical analysis. J Cell Biochem 103: 1681–1692. 17910029 10.1002/jcb.21574

[4] Kawasaki M, Iwasaki M, Koshiba T, Fujino M, Hara Y, et al (2007) Gene expression profile analysis of the peripheral blood mononuclear cells from tolerant living-donor liver transplant recipients. Int Surg 92: 276–286. 18399100

[5] Martinez-Llordella M, Lozano JJ, Puig-Pey I, Orlando G, Tisone G, et al (2008) Using transcriptional profiling to develop a diagnostic test of operational tolerance in liver transplant recipients. J Clin Invest 118: 2845–2857. 10.1172/JCI35342 18654667 PMC2483684

[6] Stewart BL, Storer B, Storek J, Deeg HJ, Storb R, et al (2004) Duration of immunosuppressive treatment for chronic graft-versus-host disease. Blood 104: 3501–3506. 15292060 10.1182/blood-2004-01-0200

[7] Pidala J, Lee SJ, Quinn G, Jim H, Kim J, et al Variation in management of immune suppression after allogeneic hematopoietic cell transplantation. Biol Blood Marrow Transplant 17: 1528–1536. 10.1016/j.bbmt.2011.03.006 21440079 PMC4497817

[8] Filipovich AH, Weisdorf D, Pavletic S, Socie G, Wingard JR, et al (2005) National Institutes of Health consensus development project on criteria for clinical trials in chronic graft-versus-host disease: I. Diagnosis and staging working group report. Biol Blood Marrow Transplant 11: 945–956. 16338616 10.1016/j.bbmt.2005.09.004

[9] Przepiorka D, Weisdorf D, Martin P, Klingemann HG, Beatty P, et al (1995) 1994 Consensus Conference on Acute GVHD Grading. Bone Marrow Transplant 15: 825–828. 7581076

[10] Van Gelder RN, von Zastrow ME, Yool A, Dement WC, Barchas JD, et al (1990) Amplified RNA synthesized from limited quantities of heterogeneous cDNA. Proc Natl Acad Sci U S A 87: 1663–1667. 1689846 10.1073/pnas.87.5.1663 PMC53542

[11] Dobbin KK, Beer DG, Meyerson M, Yeatman TJ, Gerald WL, et al (2005) Interlaboratory comparability study of cancer gene expression analysis using oligonucleotide microarrays. Clin Cancer Res 11: 565–572. 15701842

[12] Irizarry RA, Hobbs B, Collin F, Beazer-Barclay YD, Antonellis KJ, et al (2003) Exploration, normalization, and summaries of high density oligonucleotide array probe level data. Biostatistics 4: 249–264. 12925520 10.1093/biostatistics/4.2.249

[13] Irizarry RA, Bolstad BM, Collin F, Cope LM, Hobbs B, et al (2003) Summaries of Affymetrix GeneChip probe level data. Nucleic Acids Res 31: e15 12582260 10.1093/nar/gng015 PMC150247

[14] Bolstad BM, Irizarry RA, Astrand M, Speed TP (2003) A comparison of normalization methods for high density oligonucleotide array data based on variance and bias. Bioinformatics 19: 185–193. 12538238 10.1093/bioinformatics/19.2.185

[15] Tibshirani R (2006) A simple method for assessing sample sizes in microarray experiments. BMC Bioinformatics 7: 106 16512900 10.1186/1471-2105-7-106 PMC1450307

[16] Tusher VG, Tibshirani R, Chu G (2001) Significance analysis of microarrays applied to the ionizing radiation response. Proc Natl Acad Sci U S A 98: 5116–5121. 11309499 10.1073/pnas.091062498 PMC33173

[17] Subramanian A, Tamayo P, Mootha VK, Mukherjee S, Ebert BL, et al (2005) Gene set enrichment analysis: a knowledge-based approach for interpreting genome-wide expression profiles. Proc Natl Acad Sci U S A 102: 15545–15550. 16199517 10.1073/pnas.0506580102 PMC1239896

[18] Watkins NA, Gusnanto A, de Bono B, De S, Miranda-Saavedra D, et al (2009) A HaemAtlas: characterizing gene expression in differentiated human blood cells. Blood 113: e1–9. 10.1182/blood-2008-06-162958 19228925 PMC2680378

[19] Geiss GK, Bumgarner RE, Birditt B, Dahl T, Dowidar N, et al (2008) Direct multiplexed measurement of gene expression with color-coded probe pairs. Nat Biotechnol 26: 317–325. 10.1038/nbt1385 18278033

[20] Kohrt HE, Tian L, Li L, Alizadeh AA, Hsieh S, et al (2013) Identification of gene microarray expression profiles in patients with chronic graft-versus-host disease following allogeneic hematopoietic cell transplantation. Clin Immunol 148: 124–135. 10.1016/j.clim.2013.04.013 23685278 PMC8166221

[21] Brown D, Trowsdale J, Allen R (2004) The LILR family: modulators of innate and adaptive immune pathways in health and disease. Tissue Antigens 64: 215–225. 15304001 10.1111/j.0001-2815.2004.00290.x

[22] Kochi Y, Myouzen K, Yamada R, Suzuki A, Kurosaki T, et al (2009) FCRL3, an autoimmune susceptibility gene, has inhibitory potential on B-cell receptor-mediated signaling. J Immunol 183: 5502–5510. 10.4049/jimmunol.0901982 19843936

[23] Nagata S, Ise T, Pastan I (2009) Fc receptor-like 3 protein expressed on IL-2 nonresponsive subset of human regulatory T cells. J Immunol 182: 7518–7526. 10.4049/jimmunol.0802230 19494275 PMC2745186

[24] Goldstein DR, Tesar BM, Akira S, Lakkis FG (2003) Critical role of the Toll-like receptor signal adaptor protein MyD88 in acute allograft rejection. J Clin Invest 111: 1571–1578. 12750407 10.1172/JCI17573 PMC155048

[25] Porrett PM, Yuan X, LaRosa DF, Walsh PT, Yang J, et al (2008) Mechanisms underlying blockade of allograft acceptance by TLR ligands. J Immunol 181: 1692–1699. 18641305 10.4049/jimmunol.181.3.1692 PMC2840047

[26] Zhao Y, Liu Q, Yang L, He D, Wang L, et al (2013) TLR4 inactivation protects from graft-versus-host disease after allogeneic hematopoietic stem cell transplantation. Cell Mol Immunol 10: 165–175. 10.1038/cmi.2012.58 23262974 PMC4003043

[27] Aliahmad P, Kaye J (2008) Development of all CD4 T lineages requires nuclear factor TOX. J Exp Med 205: 245–256. 10.1084/jem.20071944 18195075 PMC2234360

[28] Workman CJ, Vignali DA (2005) Negative regulation of T cell homeostasis by lymphocyte activation gene-3 (CD223). J Immunol 174: 688–695. 15634887 10.4049/jimmunol.174.2.688

[29] Okamura T, Fujio K, Shibuya M, Sumitomo S, Shoda H, et al (2009) CD4+CD25-LAG3+ regulatory T cells controlled by the transcription factor Egr-2. Proc Natl Acad Sci U S A 106: 13974–13979. 10.1073/pnas.0906872106 19666526 PMC2729005

[30] Bettini M, Szymczak-Workman AL, Forbes K, Castellaw AH, Selby M, et al (2011) Cutting edge: accelerated autoimmune diabetes in the absence of LAG-3. J Immunol 187: 3493–3498. 10.4049/jimmunol.1100714 21873518 PMC3178660

[31] Jackson SH, Yu CR, Mahdi RM, Ebong S, Egwuagu CE (2004) Dendritic cell maturation requires STAT1 and is under feedback regulation by suppressors of cytokine signaling. J Immunol 172: 2307–2315. 14764699 10.4049/jimmunol.172.4.2307

[32] Shi Y, Feng Y, Kang J, Liu C, Li Z, et al (2007) Critical regulation of CD4+ T cell survival and autoimmunity by beta-arrestin 1. Nat Immunol 8: 817–824. 17618287 10.1038/ni1489

[33] Hu Z, Huang Y, Liu Y, Sun Y, Zhou Y, et al (2011) beta-Arrestin 1 modulates functions of autoimmune T cells from primary biliary cirrhosis patients. J Clin Immunol 31: 346–355. 10.1007/s10875-010-9492-4 21243522

[34] Lesley R, Xu Y, Kalled SL, Hess DM, Schwab SR, et al (2004) Reduced competitiveness of autoantigen-engaged B cells due to increased dependence on BAFF. Immunity 20: 441–453. 15084273 10.1016/s1074-7613(04)00079-2

[35] Sarantopoulos S, Stevenson KE, Kim HT, Bhuiya NS, Cutler CS, et al (2007) High levels of B-cell activating factor in patients with active chronic graft-versus-host disease. Clin Cancer Res 13: 6107–6114. 17947475 10.1158/1078-0432.CCR-07-1290 PMC2941091

[36] Sarantopoulos S, Stevenson KE, Kim HT, Cutler CS, Bhuiya NS, et al (2009) Altered B-cell homeostasis and excess BAFF in human chronic graft-versus-host disease. Blood 113: 3865–3874. 10.1182/blood-2008-09-177840 19168788 PMC2670799

[37] McLean S, Di Guglielmo GM (2010) TGF beta (transforming growth factor beta) receptor type III directs clathrin-mediated endocytosis of TGF beta receptor types I and II. Biochem J 429: 137–145. 10.1042/BJ20091598 20406198

[38] Baron C, Somogyi R, Greller LD, Rineau V, Wilkinson P, et al (2007) Prediction of graft-versus-host disease in humans by donor gene-expression profiling. PLoS Med 4: e23 17378698 10.1371/journal.pmed.0040023 PMC1796639

[39] Lu Y, Waller EK (2009) Dichotomous role of interferon-gamma in allogeneic bone marrow transplant. Biol Blood Marrow Transplant 15: 1347–1353. 10.1016/j.bbmt.2009.07.015 19822293 PMC2782586

[40] Choi J, Ziga ED, Ritchey J, Collins L, Prior JL, et al (2012) IFNgammaR signaling mediates alloreactive T-cell trafficking and GVHD. Blood 120: 4093–4103. 10.1182/blood-2012-01-403196 22972985 PMC3496960

[41] Koenecke C, Lee CW, Thamm K, Fohse L, Schafferus M, et al (2012) IFN-gamma production by allogeneic Foxp3+ regulatory T cells is essential for preventing experimental graft-versus-host disease. J Immunol 189: 2890–2896. 10.4049/jimmunol.1200413 22869903

[42] Bryceson YT, Chiang SC, Darmanin S, Fauriat C, Schlums H, et al (2011) Molecular mechanisms of natural killer cell activation. J Innate Immun 3: 216–226. 10.1159/000325265 21454962

[43] Abrahamsen IW, Somme S, Heldal D, Egeland T, Kvale D, et al (2005) Immune reconstitution after allogeneic stem cell transplantation: the impact of stem cell source and graft-versus-host disease. Haematologica 90: 86–93. 15642674

[44] Skert C, Damiani D, Michelutti A, Patriarca F, Arpinati M, et al (2009) Kinetics of Th1/Th2 cytokines and lymphocyte subsets to predict chronic GVHD after allo-SCT: results of a prospective study. Bone Marrow Transplant 44: 729–737. 10.1038/bmt.2009.80 19398965

[45] Liu Y, Zhang W (2008) Identification of a new transmembrane adaptor protein that constitutively binds Grb2 in B cells. J Leukoc Biol 84: 842–851. 10.1189/jlb.0208087 18559951 PMC2516900

[46] Choubey D (2012) Interferon-inducible Ifi200-family genes as modifiers of lupus susceptibility. Immunol Lett 147: 10–17. 10.1016/j.imlet.2012.07.003 22841963 PMC3425670

[47] Peterson KS, Huang JF, Zhu J, D'Agati V, Liu X, et al (2004) Characterization of heterogeneity in the molecular pathogenesis of lupus nephritis from transcriptional profiles of laser-captured glomeruli. J Clin Invest 113: 1722–1733. 15199407 10.1172/JCI19139 PMC420500

