# Supplementary material for: Tolerance Associated Gene Expression following Allogeneic Hematopoietic Cell Transplantation
Source: PLoS One. 2015 Mar 16;10(3):e0117001. doi: 10.1371/journal.pone.0117001 (PMC4361657; doi:10.1371/journal.pone.0117001)
Supplement: S3 File — (DOCX) [file pone.0117001.s003.docx]

**Classfier with 20 genes.**

Genes Used:

Confusion Matrix:

Accuracy

1 ( 4/ 4) 100.00% 100.00%

2 ( 4/ 4) 100.00% 100.00%

3 ( 2/ 3) 50.00% 66.67%

4 ( 3/ 3) 100.00% 100.00%

5 ( 3/ 3) 100.00% 100.00%

6 ( 3/ 3) 100.00% 100.00%

7 ( 2/ 3) 50.00% 66.67%

8 ( 2/ 3) 75.00% 66.67%

9 ( 3/ 3) 100.00% 100.00%

10 ( 3/ 3) 100.00% 100.00%

---------------------------------------------

Ave ( 2.9) 87.50% 90.00%

Confusion Matrix

(a) (b) <-- Classified As

------------------------------

14 1 (a) tolerant

2 15 (b) non-tolerant

CM Accuracy 90.62%

**Classfier with 30 genes.**

Genes Used:

Confusion Matrix:

Accuracy

1 ( 4/ 4) 100.00% 100.00%

2 ( 4/ 4) 100.00% 100.00%

3 ( 2/ 3) 50.00% 66.67%

4 ( 3/ 3) 100.00% 100.00%

5 ( 3/ 3) 100.00% 100.00%

6 ( 2/ 3) 50.00% 66.67%

7 ( 2/ 3) 50.00% 66.67%

8 ( 2/ 3) 75.00% 66.67%

9 ( 3/ 3) 100.00% 100.00%

10 ( 3/ 3) 100.00% 100.00%

---------------------------------------------

Ave ( 2.8) 82.50% 86.67%

Confusion Matrix

(a) (b) <-- Classified As

------------------------------

13 2 (a) tolerant

2 15 (b) non-tolerant

CM Accuracy 87.50%

**Classfier with 40 genes.**

Genes Used:

Confusion Matrix:

Accuracy

1 ( 4/ 4) 100.00% 100.00%

2 ( 4/ 4) 100.00% 100.00%

3 ( 2/ 3) 50.00% 66.67%

4 ( 3/ 3) 100.00% 100.00%

5 ( 3/ 3) 100.00% 100.00%

6 ( 2/ 3) 50.00% 66.67%

7 ( 2/ 3) 50.00% 66.67%

8 ( 2/ 3) 75.00% 66.67%

9 ( 3/ 3) 100.00% 100.00%

10 ( 3/ 3) 100.00% 100.00%

---------------------------------------------

Ave ( 2.8) 82.50% 86.67%

Confusion Matrix

(a) (b) <-- Classified As

------------------------------

13 2 (a) tolerant

2 15 (b) non-tolerant

CM Accuracy 87.50%

**Classfier with 50 genes.**

Genes Used:

Confusion Matrix:

Accuracy

1 ( 4/ 4) 100.00% 100.00%

2 ( 4/ 4) 100.00% 100.00%

3 ( 2/ 3) 50.00% 66.67%

4 ( 3/ 3) 100.00% 100.00%

5 ( 3/ 3) 100.00% 100.00%

6 ( 2/ 3) 50.00% 66.67%

7 ( 2/ 3) 50.00% 66.67%

8 ( 2/ 3) 75.00% 66.67%

9 ( 3/ 3) 100.00% 100.00%

10 ( 3/ 3) 100.00% 100.00%

---------------------------------------------

Ave ( 2.8) 82.50% 86.67%

Confusion Matrix

(a) (b) <-- Classified As

------------------------------

13 2 (a) tolerant

2 15 (b) non-tolerant

CM Accuracy 87.50%

**Classfier with 60 genes.**

Genes Used:

Confusion Matrix:

Accuracy

1 ( 4/ 4) 100.00% 100.00%

2 ( 4/ 4) 100.00% 100.00%

3 ( 2/ 3) 50.00% 66.67%

4 ( 3/ 3) 100.00% 100.00%

5 ( 3/ 3) 100.00% 100.00%

6 ( 2/ 3) 50.00% 66.67%

7 ( 3/ 3) 100.00% 100.00%

8 ( 2/ 3) 75.00% 66.67%

9 ( 3/ 3) 100.00% 100.00%

10 ( 3/ 3) 100.00% 100.00%

---------------------------------------------

Ave ( 2.9) 87.50% 90.00%

Confusion Matrix

(a) (b) <-- Classified As

------------------------------

14 1 (a) tolerant

2 15 (b) non-tolerant

CM Accuracy 90.62%

**Classfier with 70 genes.**

Genes Used:

Confusion Matrix:

Accuracy

1 ( 4/ 4) 100.00% 100.00%

2 ( 4/ 4) 100.00% 100.00%

3 ( 2/ 3) 50.00% 66.67%

4 ( 3/ 3) 100.00% 100.00%

5 ( 3/ 3) 100.00% 100.00%

6 ( 2/ 3) 50.00% 66.67%

7 ( 2/ 3) 50.00% 66.67%

8 ( 2/ 3) 75.00% 66.67%

9 ( 3/ 3) 100.00% 100.00%

10 ( 3/ 3) 100.00% 100.00%

---------------------------------------------

Ave ( 2.8) 82.50% 86.67%

Confusion Matrix

(a) (b) <-- Classified As

------------------------------

13 2 (a) tolerant

2 15 (b) non-tolerant

CM Accuracy 87.50%

**Classfier with 80 genes.**

Genes Used:

Confusion Matrix:

Accuracy

1 ( 4/ 4) 100.00% 100.00%

2 ( 4/ 4) 100.00% 100.00%

3 ( 2/ 3) 50.00% 66.67%

4 ( 3/ 3) 100.00% 100.00%

5 ( 3/ 3) 100.00% 100.00%

6 ( 2/ 3) 50.00% 66.67%

7 ( 2/ 3) 50.00% 66.67%

8 ( 2/ 3) 75.00% 66.67%

9 ( 3/ 3) 100.00% 100.00%

10 ( 3/ 3) 100.00% 100.00%

---------------------------------------------

Ave ( 2.8) 82.50% 86.67%

Confusion Matrix

(a) (b) <-- Classified As

------------------------------

13 2 (a) tolerant

2 15 (b) non-tolerant

CM Accuracy 87.50%
